# Reply to Comment on “Screening and Identification of Novel Ochratoxin A-Producing Fungi from Grapes”. *Toxins* 2016, 8, 333”–in Reporting Ochratoxin A Production from Strains of Aspergillus, Penicillium and Talaromyces

**DOI:** 10.3390/toxins9020066

**Published:** 2017-02-14

**Authors:** Xiaoyun Zhang, Yulin Li, Haiying Wang, Xiangyu Gu, Xiangfeng Zheng, Yun Wang, Junwei Diao, Yaping Peng, Hongyin Zhang

**Affiliations:** 1 School of Food and Biological Engineering, Jiangsu University, 301 Xuefu Road, Zhenjiang 212013, China; zhangxiaoyungu@126.com (X.Z.); wanghaiying914@163.com (H.W.); 18796002921@163.com (X.Z.); 13952967706@163.com (Y.W.); wenjun414727@163.com (J.D.); 15751011009@163.com (Y.P.); 2 Hubei Key Laboratory, Edible Wild Plants Conservation and Utilization, 11 Cihu Road, Huangshi 435002, China; liyulin7226@163.com; 3 School of Grain Science and Technology, Jiangsu University of Science and Technology, 2 Mengxi Road, Zhenjiang 212003, China; sdxyugu@126.com

We sincerely thank you and Dr. Giancarlo Perrone, Antonio F. Logrieco and Jens C. Frisvda for the suggestions and concerns raised with regard to our paper entitled “Screening and Identification of Novel ochratoxin A-Producing Fungi from Grapes” which was published in *Toxins*. We fully understand that their concerns are aimed towards improving scientific research for the broad readership of the scientific community. First and foremost, in response to the eight steps outlined by Dr. Perrone et al., we want to state that pure culture of the isolates were used. We also want to emphasize that proper media and culture conditions were used and adhered to throughout the assays. These imply steps 1 and 2 were strictly followed. Furthermore, the fungi species were identified on the basis of *ITS* sequencing and other conserved genes (*BenA* and *RPB2*) proposed by Dr. Perrone et al. in the comments.

On the issue of accession, we preserved the fungi O1, MQ-5 and MB1-1 in China General Microbiological Culture Collection Center (CGMCC) with serial numbers as follows: O1 = 3.15508, MQ-5 = 3.15509 and MB1-1 = 3.15653. Q3 was identified as the same strain as O1, so it wasn’t previously preserved in CGMCC. We are currently preparing to send Q3 to CGMCC or China Center for Type Culture Collection (CCTCC) for preservation as soon as possible.

Furthermore, Dr. Perrone el al. expressed concern with the analytical chemical detection of the presence of OTA in the culture using only HPLC-FLD (fluorescence detection) as being inadequate as it is possible that other fluorescing compounds with the same retention time could be mistaken for OTA. We do agree with this assertion. However, we screened out the five OTA-producing fungi from the 139 isolates by the UV fluorescence method combined with 25%–28% ammonia fumigation (characteristic green fluorescence) and HPLC-FLD retention time as compared to a standard. The probability of other fluorescing compounds with the same fluorescence characteristics and retention time produced by the same strain in this condition was very low. We also agree with the comment that it is good to confirm the OTA by other measures, and to check the presence and expression of gene cluster involved in the OTA biosynthetic pathway in order to authenticate if indeed the toxin produced was OTA. In line with this, we would consider investigating it further for the purposes of verification.

In conclusion, it is a good proposal for subsequent research work that the eight steps put forward by Dr. Perrone et al. need to be investigated fully before drawing the conclusion as to whether a particular strain of fungus can produce a certain toxin. We want to state that due diligence was made to ensure that the fungi were properly identified according to the methods stated in the paper. Indeed, our research team relies on scholarly articles published by others; however, there might be a need to conduct further research to verify the claim that those fungi can produce OTA. We will submit and publish our results when we have finished the experiments. We sincerely appreciate your time and professionalism. Once again, thank you and Dr. Giancarlo Perrone, Antonio F. Logrieco and Jens C. Frisvda for the good suggestions delivered as a friendly reminder.

